# A multisource approach to health care use: concordance between register and self-reported physician visits in the foreign-born population in Finland

**DOI:** 10.1186/s12874-022-01780-w

**Published:** 2022-12-02

**Authors:** Regina García-Velázquez, Valentina Kieseppä, Eero Lilja, Päivikki Koponen, Natalia Skogberg, Hannamaria Kuusio

**Affiliations:** Finnish Institute for Health and Welfare, Mannerheimintie 166, PL/PB/P.O. Box 30, FI-00271 Helsinki, Finland

**Keywords:** Survey methods, Registers, Validity, Health care use, Foreign-born population

## Abstract

**Background:**

Reliable information on the use of health services is important for health care planning, monitoring and policy. It is critical to assess the validity of the sources used for this purpose, including register and survey-based data. Studies on foreign-born populations’ health care use have usually implemented either survey or register data. The concordance of such data among groups of different cultural background remains largely unknown. In this study, we presented an approach to examine routinely how survey and register-related characteristics may explain disagreement found between the two information sources.

**Methods:**

We linked register- and survey-based data pertaining to the Finnish Register of Primary Health Care general physician visits and the Survey on Well-Being among Foreign Born Population (FinMonik, 2018–2019), a nationally representative survey. The sample comprised *n* = 5,800 informants for whom registered general physician visits were tracked in the 12-month period preceding their participation in the survey. Cohen’s kappa was used as measure of multisource concordance, hierarchical loglinear models for the association between single predictors and multisource discrepancy, and a logistic regression model for examining source-related predictors of source discrepancy. Survey weights were used in all sample analyses.

**Results:**

Source concordance was poor. When dichotomizing general physician visits (zero vs one or more), 35% of informants had reported one or more visits while none were found from register. Both register- and informant-related predictors were associated to this discrepancy (i.e. catchment area, private health care use, inability to work, region of origin and reason for migration).

**Conclusions:**

We found high discrepancy between the reported and the registered physician visits among the foreign-born population in Finland, with a particularly high number of reported physician visits when none were found in the register. There was a strong association between the specific catchment area and mismatch, indicating that both register under-coverage and survey over-report are plausible and may coexist behind the discrepancy. However, associations of informant’s characteristics and mismatch were less pronounced. Implications on the validity of medical information sources are discussed.

## Introduction

Reliable information on the use of health services is essential for health care planning, monitoring, and policy. Often information on health care use is gathered via surveys. With surveys it is possible to collect information over many different factors simultaneously, and survey data are often easier to attain than register data due to the latter’s strict requirements for collection. In contrast, survey information is susceptible to bias which can be caused by problems in recalling, underreporting of visits which might be associated with stigma, or imprecise formulation of the items measuring the service use [1]. Inaccuracy in reporting typically increases with longer recall periods and higher actual utilization frequency [1]. Unlike register data, survey data is also very expensive to collect [2] and brings about social desirability concerns.

Register data on health service use is often seen as the “gold standard” to which survey data is compared against. Registers are powerful health information systems because of being timely, standardized, and free from informant-related challenges. The knowledge obtained from medical registers is an important asset in decision making, with crucial implications for public health. Medical records are routinely used for multiple purposes, such as health monitoring, evaluation and ranking of health care service performance, or as criterion for assessing quality of other methods for collecting health information, such as measurement of rating scales in research (e.g., in ROC analyses) and health care outcomes as self-reported in surveys (e.g., visits to health professionals, medical conditions diagnosed by doctors, use of medication, surgeries or health interventions, etc.).

Sometimes, registers are not available for use. Multisource studies offer naturally more reliable information than single-source studies, and are often used to validate other sources of information (i.e. survey-based) in concordance studies. Many registers undergo studies on the quality and validity of their data [3–5], and their properties are known. However, despite standardization register data can have inaccuracies or gaps in coverage [6–8]. Further, information about the timeliness and coverage of registers is sometimes lacking or obsolete, posing challenges on the validity of conclusions made [9, 10]. Failing to account for register-related properties in multisource concordance studies implies the (sometimes untested) assumption that the register is unbiased and complete (i.e. and thus benchmarked against survey-based information, to which bias is potentially attributed). For such an assumption, quality of the registers in use ought to be known and inspected regularly.

The Finnish Hospital Discharge Register (Hilmo), which is maintained by the Finnish Institute for Health and Welfare (THL), covers data from all patient encounters within the public hospital inpatient care (since 1967) and outpatient care (since 1998). Since 2011 the Hilmo register has also included the register of primary healthcare visits (avoHilmo) to cover all encounters in public primary health care centers [11]. The information on visits to health care are automatically collected from the patient records and transferred to THL. As hospital districts in Finland use different medical records systems, there might be some variation in the recording and transferring of the information across different areas. Since 2021, avoHilmo also includes visits to occupational health care, but these visits are not comprehensively covered during our study period. The completeness of the Hilmo register is known to be good [3], but the completeness of the avoHilmo has not been systematically studied.

Many international studies have compared self-reported and register data on health care use, typically with the intention of validating survey data against the benchmarked register data [12–16]. The studies have often focused on specific groups, such as the elderly [8, 17–19] or groups with a specific diagnosis [20–23]. Hospitalizations have usually been found to be self-reported with higher accuracy [16, 24, 25], whereas there is less accuracy in self-reports of emergency department visits [22, 24] and physician visits [24–26]. In general, studies which have focused on the concordance between self-reported and registered physician visits have typically found a tendency towards under-reporting in surveys [14, 21, 23, 27], or have provided mixed results [8, 13, 16, 17, 20].

Older age has been associated with underreporting health care use [12, 25], and poor health status to overreporting [8, 12, 14]. The actual number of visits has been associated with both under- and over-reporting, suggesting there is no systematic direction of self-report bias as a function of number of registered visits [12, 27].

Studies on foreign-born populations’ health care use have usually used either survey or register data, and the concordance of self-reported and registered health care visits among groups of different cultural background has, to our knowledge, only been addressed in one study before. This Dutch study found that agreement between self-reported and registered health care use was slightly lower for immigrants as compared to the Dutch-born population, particularly for immigrants from Turkey and Morocco, but there was no systematic bias towards either direction [15].

The purpose of this study was twofold: methodological and substantial. First, we proposed a general approach to inspect multisource (dis)agreement while examining each source critically (i.e. by taking into account how each source’s characteristics are associated with potential discrepancies between sources). By this, we abandoned the common practice of assuming that measures obtained by informants must contain error whenever discrepancies are found in registers. Second, we used this approach to answer to the specific question of concordance over general physician visits between a national survey of foreign-born population and the primary health care register from Finland. Informant-related (age, sex, employment status, self-reported health condition, self-reported visits to occupational physician/private physician, reason for migration and region of origin) and health care register-related characteristics (specific catchment area) were considered.

## Methods

### Sample

This study utilizes data from the Survey on Well-Being among Foreign Born Population (FinMonik, 2018–2019), which is so far the most extensive survey carried out among the foreign-born population in Finland. It comprises a wide range of self-reported data, including information on the respondent's health, well-being and access to care.

The sample was obtained in March 2018 from the population register maintained by the Digital and Population Data Service Agency. The sample was drawn using the following criteria: (1) the respondent’s country of birth must be other than Finland; (2) both parents or the only known parent of the respondent must have been born abroad; (3) the respondent must have lived in Finland for at least for a year at the time of sampling; (4) the respondent must be aged 18–64 at the time of sampling, and (5) the respondent must not have come to Finland through adoption. An amount of 13,650 individuals were selected by means of stratified random sampling as total population of the study. A mixed-methods approach (an electronic questionnaire, a paper questionnaire and phone interviews) was used to facilitate study participation. All response types included, the response rate was 53.1% (*n* = 6,836 participants). Sample weights were calculated to account for non-participation and calibrated according to age, sex, stratum (area of residence), country group and education. The sample and related methodology are well documented elsewhere [28]. Register-based data from several national registers on sociodemographic variables (age, sex, country of origin and catchment area) access to health care was available to us and linked to the survey data by using the personal identity number provided to all permanent residents in Finland.

The analytic sample of this study is comprised of those participants who (a) answered to the survey and (b) consented to linking their answers to official health registers (*n* = 5,800). Sociodemographic variables were available via linkage from the Digital and Population Data Services Agency. The Register of Primary Health Care visits (AvoHilmo) covers activities of outpatient primary health care for purposes of statistics, research, development, and planning. The register is also used in the monitoring of, for example, infectious diseases, injuries, and health examinations as well as in other tasks of which the Finnish Institute for Health and Welfare is responsible of. For more information on the AvoHilmo register, see [29].

### Variables and materials

The survey-based information used for concordance assessment was based on the following item: “*How many times in the past 12 months have you seen a doctor, public health nurse or a nurse in an appointment or at your home because of an illness you have or had (or because of pregnancy or childbirth)? a. At a health centre (no dental appointments), I saw a doctor __ times*”. The equivalent information was tracked from register records by limiting the number of visits to the twelve-month period preceding the day the survey was completed. Apart from professional being a physician, other criteria for drawing registered visits were: pertaining to primary care, taken place at health care center, being an individual visit (as opposed to group session or family visit), and visit not been cancelled.

Additional to number of general physician visits as self-reported and registered records, we used an array of variables for characterizing concordance between survey- and register-based data. The informant-related variables were: *age* (register-based and coded into three groups: 18–29, 30–49, 50–64), sex (register-based: male or female), *region of origin* adapted from the United Nations Standard country or area codes for statistical use [30] (register-based; coded into nine groups: Russia and the former Soviet Union; Estonia; Europe (excl. Russia and Estonia), North America and Oceania; Middle East and North Africa; Africa (excl. North Africa); Southeast Asia; East Asia; South and Central Asia; and Latin America), self-reported *reason for migration* to Finland (coded into five groups: own job, own studies, family reasons, asylum or international protection, and being Ingrian Finn or returnee), self-reported economic activity (working, student, other), self-reported *chronic condition* (binary), self-reported *diagnosis of a mental health disorder* in 12-month period (coded into no, one diagnoses, more than one diagnosis), self-reported *diabetes* diagnosed by doctor in 12-month period (binary), self-reported *hypertension or heart disease* diagnosed by doctor in 12-month period (binary), self-reported *asthma* diagnosed by doctor in 12-month period (binary), self-reported *ability to work* (coded into three categories: completely able, partly unable, completely unable), self-reported *visits to private physician,* meaning occupational healthcare and/or any form of self-paid visit to physician in preceding 12-month period (binary, coded into zero and one or more).

Last, we included *catchment area* to account for register-related variation upon multisource agreement. Catchment area is reliably collected in registers and may show systematic variation due to the use of different medical record systems across regions in Finland. The variable was coded into six categories: Hospital District of Helsinki and Uusimaa (hereinafter referred to as HUS, the hospital district in the capital region analyzed separately here due to the area’s high population density), Southern Finland (to which HUS belongs), Western Finland, Central Finland, Northern Finland.

### Data analysis

The distribution of both self-reported and register-based general physician visits were preliminary inspected as count variables and finally dichotomized into zero versus one or more visits. Sample-weighted Cohen’s kappa was estimated as measure of agreement between both information sources that accounts for chance agreement and ranges from -1 to 1. A moderate level of agreement corresponds to values between κ = 0.60 to κ = 0.79 [31]. The level of agreement was also inspected via percent agreement in two- and three-way cross tabulation (note this approach does not account for chance agreement, which is considerably high in dichotomous variables).

As an exploratory analysis, a series of hierarchical loglinear models [32] were fit for each of the variables of interest crossed against the agreement over general physician visits (resulting in thirteen series of three-dimensional hierarchical loglinear models). The purpose of these models was to inspect in a univariate fashion whether the predictor (e.g., age group) was associated with the observed distribution of pairwise agreement. The series of loglinear models were fit from null (all variables independent) to saturated (second-order association between the three variables). Predictor-based differences in concordance of self-reported and registered general physician visits were measured by the saturated model. Note that these hierarchical models contain all main effects and first-order interactions. Model selection targeted most parsimonious of the well-fitting models for each predictor and was based on deviance values and chi-square tests of goodness of fit (GOF) for absolute and relative model selection (of which null hypothesis is that the model fits the observed data, and thus *p* > 0.05 suggests well-fitting models).

Finally, we fitted a logistic regression model predicting whether the participant had reported having visited a general physician while no record was found on registers (s_yes_,r_no_, also referred to as mismatch or over-report henceforth). We chose this event as our outcome of interest as we found it to be markedly high for a multisource disagreement. The analytic sample for this regression model comprised participants with the following bivariate pattern: s_yes_,r_no_, s_yes_,r_yes_, and s_no_,r_yes_, and consisted of *n* = 2,447. The category s_no_,r_no_ was of no relevance to our regression model, since we were interested in use of health care. All variables were included as predictors in the model in order to estimate fully adjusted coefficients. The model was assessed by means of Nagelkerke’s pseudo R^2^ and model diagnostics conducted to inspect influence of extreme cases and model assumptions. Because the customary odds ratios (OR) in logistic models have limitations [33], we present along the Average Marginal Effect (AME) of every predictor in the regression model, as recommended [34]. AME can be read as the *average change in probability* for the outcome to occur when the predictor or increases by one unit (switches category from baseline, in case of categorical predictor). Average change implies that AMEs are estimated by conditioning on the observed values of all other covariates for each observation. AMEs get values [-1,1], with negative indicating decrease and positive values increase in probability. Confidence intervals and significance tests of AMEs were also included in this study.

The stratified random sampling structure of the data was accounted for in all stages of data analysis by using sample weights and their stratum structure. All analyses were performed in R [35] and we used the packages *survey* [36] and *margins* [37].

## Results

**Fig. 1:**
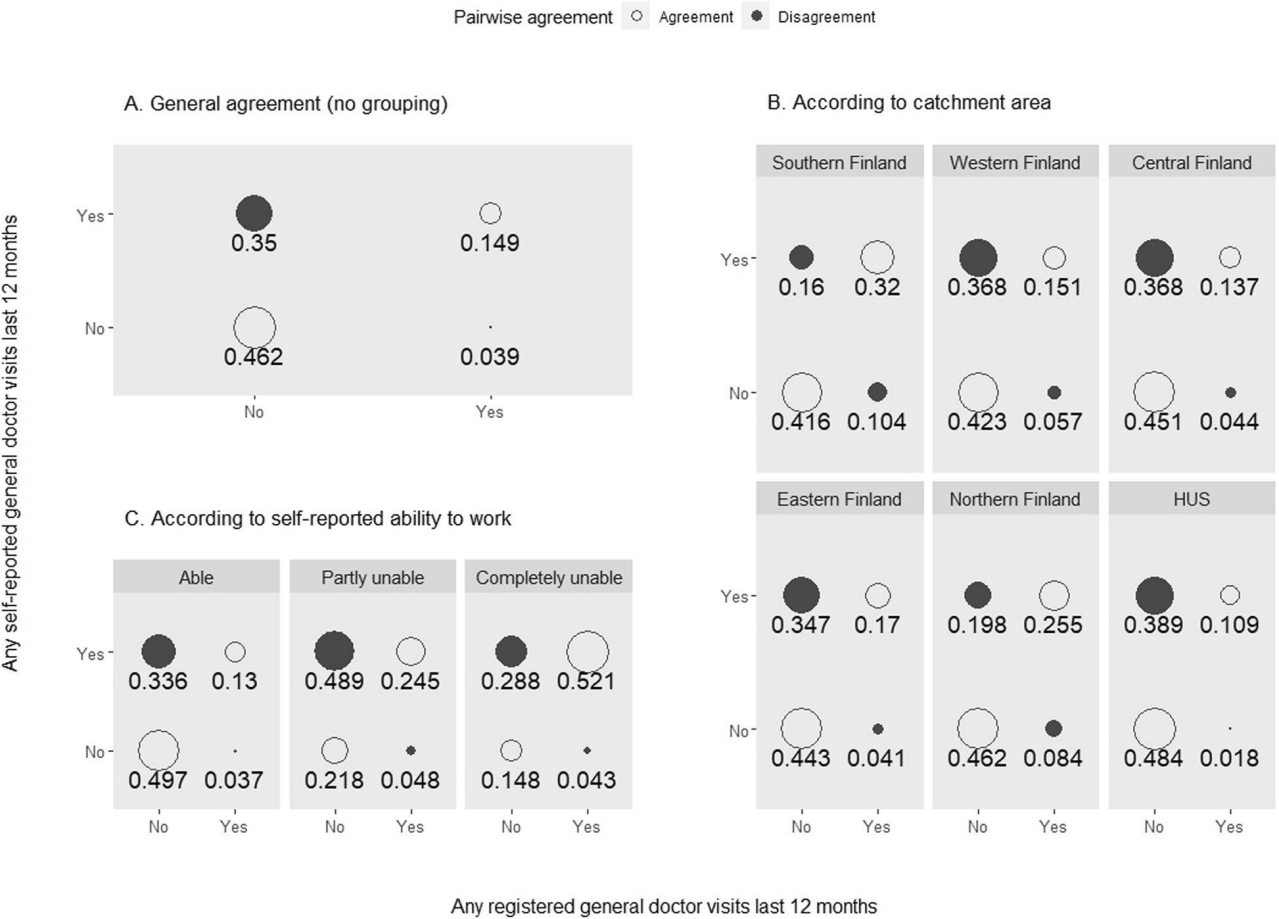
Sample-weighted agreement between self-reported and registered general physician visits during the previous 12-month period to the FinMonik survey

A total of 3,478 valid, registered general physician visits, were found and linked to 1,506 participants. Most of the participants of the survey had null record track (74.03%), while 75.8% of them had self-reported visits (*n* = 4,394). Sample characteristics can be found in Table 1. For number of visits (as count variable), the agreement for zero visits was 46.2% and 5.2% for one visit, while agreement of two and above number of visits was negligible and added up to a total of 51.4% agreement. Of the participants reporting visits to general physician, only 30.0% had one or more corresponding record in registers. Of the participants reporting visits while zero registers were found, 36.7% reported only one visit and 26.1% reported two. Of the participants with at least one record in registers, 7.8% did not report any general physician visit in the survey. Dichotomizing the variables (none vs. one or more visits) naturally increased the total agreement over general physician visits up to 61.1% (dichotomized variable agreement shown in Fig. 1A). Sample-weighted Cohen’s kappa for dichotomous agreement was κ = 0.221 (s.e. = 0.015), which is considered minimal.

**Table 1:** Sample-weighted descriptives of the participants in FinMonik survey^a^

**Variable**	**Groups**	**N (raw)**	**%**	**Self-reported visits (mean)**	**Registered visits (mean)**
Gender^b^	Female	2431	50.6	2,1	0,6
	Male	1963	49,4	1,3	0,3
Age^b^	18–29	1011	24.6	1,3	0,4
	30–49	2463	57.6	1,7	0,4
	50–64	920	17.8	2,1	0,5
Occupation^c^	Work	2100	57.4	1,6	0,5
	Studies	611	18.4	2,1	0,4
	Other	802	24.3	1,6	0,4
Catchment area^b^	Central Finland	617	9.7	1,6	0,4
	Eastern Finland	852	8.4	1,5	0,5
	Hospital District of Helsinki and Uusimaa (HUS)	816	54	1,8	0,3
	Northern Finland	849	6.1	1,3	0,7
	Southern Finland (Other than HUS)	609	8.2	1,3	1,1
	Western Finland	651	13.5	1,9	0,5
Hypertension or heart disease diagnosis^c^	No	3644	89,5	1,6	0,4
	Yes	471	10.5	2,1	0,7
Astma diagnosis^c^	No	3821	94,1	1,5	0,4
	Yes	208	5.9	3,6	0,7
Diabetes diagnosis^c^	No	3849	95,6	1,4	0,4
	Yes	179	4.4	5,4	1,0
Mental health diagnosis^c^	No	3897	88.3	1,5	0,4
	One diagnosis	362	8.5	2,4	0,7
	More than one diagnoses	135	3.2	4,6	1,3
Chronic condition^c^	No	2874	68,8	1,2	0,4
	Yes	1483	31.2	2,7	0,6
Region of origin^b^	East Asia	213	5.5	0,8	0,2
	Estonia	401	11	1,5	0,4
	Latin America	122	4.3	1,7	0,4
	Middle East and North-Africa	587	14.5	2,9	0,7
	Rest of Africa	211	7.6	2,2	0,5
	Rest of Europe. North-America and Oceania	869	20.2	1,4	0,4
	Russia and Soviet Union	1352	22	1,3	0,5
	South and Central Asia	213	6.5	1,4	0,2
	Southeast Asia	426	8.4	1,8	0,3
Reason for migration^c^	Asylum or international protection	482	11.8	2,7	0,8
	Being Ingrian Finn or returnee	427	8.2	1,5	0,4
	Family reasons	2009	44.2	1,8	0,5
	Own job	862	21.4	1,5	0,3
	Own studies	552	14.4	1,0	0,2
Visits to occupational healthcare and/or private physician^c^	No	2314	57,7	1,0	0,4
	Yes	1482	42.3	2,0	0,3
Ability to work^c^	Able	3727	88	1,4	0,4
	Completely unable	92	1.8	3,5	0,9
	Partly unable	540	10.2	7,6	1,6

^a^The prevalences are calculated from the survey participants for whom information on self-reported physician visits was available and linked to registers

^b^Information obtained from registers

^c^Self-reported in survey

Two of the predictors showed statistically significant association at second order in well-fitting models: catchment area (overall model GOF: deviance = -2.93e-13, *p* = 1 with 0 residual df) and ability to work (overall model GOF: deviance = 4.78e-13, *p* = 1 with 0 residual df). As can be seen in Fig. 1B, some catchment areas presented more mismatch than others, and this was clearer for the area corresponding to HUS. Figure 1C shows that agreement matched the direction of the variable ability to work, while mismatch was highest for the category “partly unable”. The rest of the hierarchical models fitted, corresponding to all other background variables, either obtained unacceptable fit (i.e. GOF test at *p* < 0.05 in all models, for the following variables: reason for migration to Finland, region of origin, chronic condition, asthma), or reached best fit without including second-order interaction (i.e. suggesting the variable did not have a role in the pairwise agreement of reported-registered general physician visits; this was the case for the following: age group, sex, occupation, mental health diagnosis, hypertension or heart disease, diabetes, visit to occupational/private physician).

**Fig. 2:**
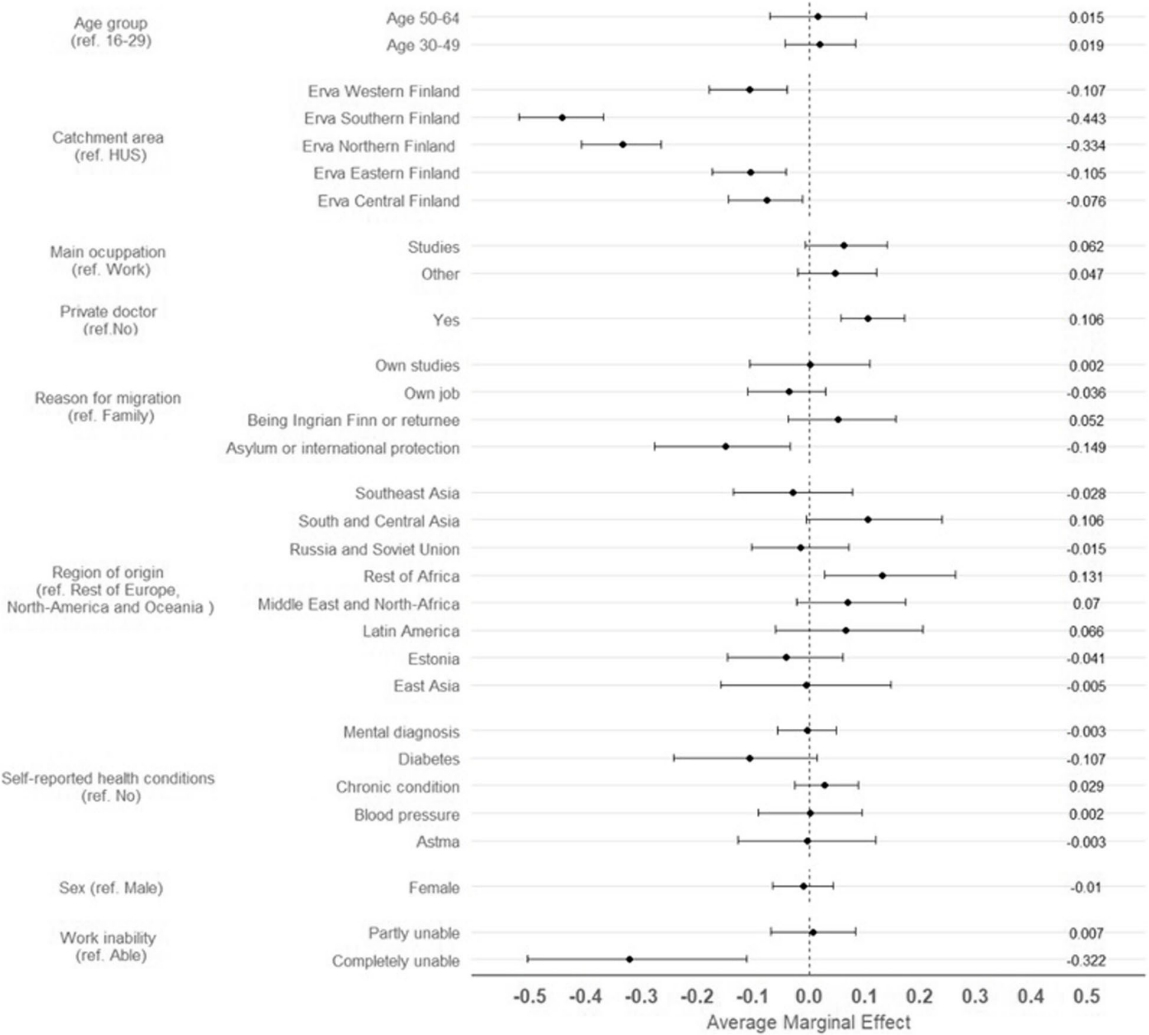
Average Marginal Effects of the logistic model predictors over mismatch between survey- and register-based information over general physician visits in the FinMonik study

The logistic regression model revealed independent variable associations while adjusting for covariates (Fig. 2). Catchment area remained a relevant predictor: all other areas than HUS were significantly less prone to mismatch. Also having arrived to Finland as refugee or seeking for asylum, and reporting to be completely unable to work showed reduced likelihood of mismatch. Predictors of mismatch were having visited a private general physician in the same time period and being Africa the country of origin (excluding North-African countries). Nagelkerke’s pseudo R^2^ was 0.187. Model diagnostics revealed that the model correctly predicted 64.5% of the observations and was more effective at predicting mismatch cases (77.5% of mismatch cases correctly predicted).

We conducted post hoc analyses to check whether the specifications of the register record search could explain the high mismatch against self-reported data (i.e. to investigate whether the lack of registered visits, as compared to self-reported, stems for mispecified search of records). All filters were excluded from the search (profession code, place, type of visit, etc.) except for time of the appointment. A total of 39, 848 registered appointments were found, corresponding to all registered visits in the whole AvoHilmo taking place within one year until participating in the survey. The sample-weighted percentage agreement for this extended register-based search was 61.3% and the kappa coefficient was κ = 0.222 (s.e. = 0.020), which is considered minimal and is practically equivalent to that of our main analyses.

## Discussion

In this study, we investigated the level of concordance between registers and self-reported general physician visits in the foreign-born population of Finland. Instead of assuming registers as benchmark, characteristics of both survey informants and registers were looked into. We found a high discrepancy between the two sources, with 35% of the informants self-reporting general physician visits when none were found in the register. The level of under-reporting was substantially lower (3.9% did not report any physician visits when at least one visit was registered). Over-reporting was nine times more prone to happen in our data than under-reporting (35% against 3.9%), thus we examined this bias in more detail and referred to it generally as mismatch.

Large discrepancies in concordance reflect issues with reliability and/or validity. In principle, systematic measurement error stemming from any of the sources is possible. Still, it is customary to link discrepancies to survey issues and benchmark medical registers. In cases where the properties of the medical registers are not well known, it is critical to consider both sources, as it was demonstrated in this study. We used commonplace methodology, but took the unconventional approach of looking simultaneously into register- and survey-related effects over the apparent over-reporting of general physician visits. Had we failed to take regional variation into account (a simple, yet good indicator of within-register heterogeneity), our results could severely reflect omitted-variable bias and our discussion focus only on the conspicuous over-reporting by the study population, as explanation alone for the mismatch in concordance. According to our findings, register under-coverage and survey over-report are both plausible and may coexist behind the large mismatch. We will discuss our findings by integrating both the informant- and register related aspects together in the following paragraphs.

Reporting at least one visit while none were registered was strongly associated with the specific catchment area of the participant. Living in any other than HUS specific hospital district (which covers the Finnish capital region) made mismatch less likely. In comparison to the HUS area, mismatch was particularly unlikely in the catchment areas of Southern and Northern Finland. Mismatch was slightly more likely if the participant was from Africa exl. North Africa as compared to being from Rest of Europe, North America or Oceania, and less likely if the reason for migration had been seeking asylum as compared to migrating for family reasons. Being completely unable to work made mismatch less likely.

There are several indicators that our finding of low concordance between the two sources might not be completely explained by inaccuracies in the survey data. First, there was a markedly high number of informants in our sample for whom no registered physician visits were found: 80.9% of the sample did not have any registered physician visits during the study period. Even though we only included public primary care visits and our sample consists of working age adults, it still seems like a low proportion in comparison to typical averages of yearly physician visits in OECD countries [38]. Second, the specific catchment area was strongly associated with likelihood of mismatch, indicating the possibility that there may be some degree of inaccuracy in the registering system (this involves different processes like record coding or register transferring among organizations). And third, such a high level of overestimation by the informants would be quite inconsistent with previous research [14, 21, 23, 27]. For these reasons it seems likely that the register data has inaccuracies or gaps, and the discussion of other associations should therefore be viewed critically.

We found that reporting private and/or occupational physician visits was positively associated with mismatch. This could be linked to either recalling bias – having had some physician visits, it can be difficult to remember whether they were public or private – or misunderstanding the question and thinking of all physician visits rather than just public ones. Actual health care utilization has been linked with more inconsistent reporting in previous studies as well [12, 27].

Poor health status has been somewhat consistently found to predict overreporting of health care use in surveys [8, 12, 14]. These studies have used counts of health care visits to define overreporting, so the results are not quite comparable to ours, but it is still worth noting that we did not find any effect of reporting any specific health condition on mismatch. However, being completely unable to work made mismatch less likely. Inability to work indicates serious health problems, which are likely to lead to physician visits, and possibly this variable reflects serious health problems in our working age population more adequately than the adjusted health conditions.

The lower likelihood of mismatch among the individuals who are completely unable to work might be due to higher actual physician utilization and thus higher likelihood of having at least one registered and reported visit. In addition, these individuals are less likely to use occupational health care, and less likely to be able to afford private health care, so there are less chances of misremembering which health care system had been used.

Finally, we found some associations between mismatch and migration-related characteristics. Having migrated as a refugee made mismatch less likely. As we know that individuals who have migrated to Finland as refugees have difficulties finding employment [39], it seems possible that they are less likely to use occupational or private health care as compared to other migrant groups. Thus, they might be less likely to incorrectly report private or occupational health service use as public health care use, leading to lower likelihood of mismatch. These variables were, however, adjusted for in our analyses and thus there seems to be an independent association between migrating as a refugee and mismatch of public physician use in Finland.

Having migrated from Africa exl. North Africa made mismatch more likely. To our knowledge only one previous study has examined differences in concordance between survey and register data among different ethnic groups [15], and they found no systematic differences in reporting. We retain from further speculations of the possible explanations of the pattern found here, as literature on health service use across different ethnic groups is very limited.

There is a general characteristic of the Finnish health care system that may play a role in mismatch found in our study. In case of presenting a health complaint, it is highly likely that the public health care user has a first appointment with a nurse, who will advise and decide to what kind of doctor the patient should be referred to if necessary. In Finland, the nurse can have a more independent role in primary health care in comparison to other countries and a visit to a nurse might completely replace the physician visit. Additional to recalling difficulties, it is possible that persons migrating to Finland are not aware of this practice and believe having met a physician. Our survey items do query separately about appointments with nurses and physicians, but it might be too much to ask for in a retrospective setting. Nonetheless, we examined this possibility by removing all register filtering criteria (except for timing of the appointment) as a post hoc analysis. The agreement between self-report and register data was virtually the same as in the original results (in terms of percentage agreement and kappa coefficient), and so there is no evidence to consider that recalling bias on the professional (physician, nurse, or any other) could explain the mismatch in our study. More generally, the results of the post hoc analysis suggest that the high degree of mismatch was unlikely due to issues related to data filtering (e.g. too narrow scope of filters, recalling bias on appointment characteristics other than time, or variables left empty in records that were consequently excluded for having missing information in key filtering criteria).

We identify two informant-level cases for mismatch not covered by our analyses: (1) misremembering the time of the appointment, (2) that some of the respondents might have used health care in their country of origin and reported these physician visits. A previous study on Russian immigrants in Finland showed that in the preceding 12 months, 15% of the informants had visited a physician in Russia [40]. However, Russian health care was typically used in parallel with Finnish health services.

Finnish general population survey studies have contained health care and disease queries in a very similar format. There is some evidence suggesting that register sources are incomplete and not adequate for estimating prevalence of certain diseases and healthcare conditions [41, 42], but there is no study published to this date on the agreement between survey and register data of Finland with respect to general physician visits. Inspecting data quality of the AvoHilmo register through general population studies is essential for understanding our results in context and making consequent recommendations, particularly in what comes to recording practices in specific catchment areas and/or up to what degree of detail is reasonable and cost-effective to query information about health care use in survey studies (e.g. recalling difficulties introducing important noise on number of times one has met a nurse or a physician in the last 12-month period). All in all, the constraining level of source disagreement on the simplest indicator of healthcare use (i.e. dichotomized as “went to public doctor”) urges to improve consistency. The efforts may target local monitoring, for instance by issuing reminders after a time interval of low or no electronic record updates, or by publishing a periodic report allowing region-level data on coverage and quality of electronic health records. Initiatives to simplify the task of completing electronic records by professionals are worthy, as it is any other way to improve the reliability of measurement in general.

### Strengths and limitations

A considerable strength of our study is the unique setting, where we were able to link the comprehensive, nation-wide population data on the foreign-born population in Finland to health care register covering all visits to public primary health care centers. In addition, we approached the lack of agreement as stemming not only from bias on the side of health care users (as it is common in the literature), but also from the health care register itself, by using the specific catchment area as proxy for register-level systematic variation.

There are some limitations associated with this study. First, the lack of supporting evidence of equivalent studies with Finnish general population, which limits our making further conclusions over how much of our results are explained by the specific population studied here.

Second, data missing in population surveys does not occur randomly. Precisely those for whom public health care is most important could have abstained from participating, as it is well known that there is social inequality in research and survey participation [43, 44]. The use of sampling methodology (i.e. stratified random sampling) and sampling weighting in our study aims at compensating for this issue, but it is still a limitation worth taking into account.

Third, knowing that the foreign-born population in Finland varies in terms area of residence (e.g. those coming from neighbor countries live closer to the borders, and metropolitan areas comprise higher density of foreign-born population than rural areas), it would be useful to adjust for such modifying associations via interaction effects. Unfortunately, sample size limitations hindered model computation. In consequence, this study is limited to main effects only.

Finally, the retrospective design of our study presents a limitation. A adequately designed study on the multisource agreement between self-reported and register data would include a prospective design where users could mark down their health care visits as they take place and include detailed information on the appointments. Instead, recall bias poses a greater limitation to retrospective studies. This is particularly visible in our study through the statistically significant association of self-reported private doctor visits with mismatch. Recall inaccuracies and/or misunderstandings are more likely in a country like Finland, were occupational, self-paid and public healthcare are used simultaneously by many citizens. While this poses a limitation to the study of healthcare use in general, we consider it is a strength of this study to query for this information via survey, private healthcare use is lacking completely or partially from most healthcare registers.

## Conclusions

We found high discrepancy between self-reported and registered physician visits among the foreign-born population in Finland, with a particularly high number of self-reported physician visits when none were found in the registers. The magnitude of the discrepancy and the strong association between the specific catchment area and mismatch suggest possible inaccuracies in the health care register. It proved central to adjust for self-reported private healthcare visits. We recommend collecting information about the co-existing forms of healthcare use when studying healthcare use in general, and multisource agreement in particular. Yet, our results should be replicated with the general population of Finland, so that conclusions over the mechanisms behind our findings could be made. Our results did not show strong associations between migration-related characteristics and mismatch.

The results in this study suggest that register data per se is not unquestionable. Failing to collect valid evidence in health may hinder efforts to achieve better and more equitable health outcomes. All sources of information shall be approached equally critically. If quality and timeliness of the medical register at use is unknown, there are methodologies at hand to study their validity also in the context of applied research, as illustrated in this study.

## Data Availability

The data that support the findings of this study can be obtained from THL but restrictions apply due to data protection regulations, ethics approval of the surveys and the subjects` consent to participate. Thus, the data are not publicly available. The survey data are however available for request from the survey website: https://thl.fi/en/web/thlfi-en/research-and-development/research-and-projects/solid-knowledge-base-and-research-based-information-about-integration-finmonik2-
